# An Evaluation of Association between a Novel Hippocampal Biology Related SNP (rs7294919) and Schizophrenia

**DOI:** 10.1371/journal.pone.0080696

**Published:** 2013-11-22

**Authors:** Jiewei Liu, Shusuke Numata, Masashi Ikeda, Yuichiro Watanabe, Xue-bin Zheng, Xiongjian Luo, Makoto Kinoshita, Ayako Nunokawa, Toshiyuki Someya, Tetsuro Ohmori, Jin-xin Bei, Siow-Ann Chong, Jimmy Lee, Zhiqiang Li, Jianjun Liu, Nakao Iwata, Yongyong Shi, Ming Li, Bing Su

**Affiliations:** 1 State Key Laboratory of Genetic Resources and Evolution, Kunming Institute of Zoology, Chinese Academy of Sciences, Kunming, Yunnan, China; 2 Department of Psychiatry, Course of Integrated Brain Sciences, Medical Informatics, Institute of Health Biosciences, The University of Tokushima Graduate School, Kuramoto-cho, Tokushima, Japan; 3 Department of Psychiatry, School of Medicine, Fujita Health University, Toyoake, Aichi, Japan; 4 Department of Psychiatry, Niigata University Graduate School of Medical and Dental Sciences, Niigata, Japan; 5 Human Genetics, Genome Institute of Singapore, A*STAR, Singapore; 6 Flaum Eye Institute and Department of Ophthalmology, University of Rochester, Rochester, New York, United States of America; 7 Institute of Mental Health, Singapore; 8 Bio-X Institutes, Key Laboratory for the Genetics of Developmental and Neuropsychiatric Disorders (Ministry of Education), Shanghai Jiao Tong University, Shanghai, China; 9 University of Chinese Academy of Sciences, Beijing, China; University of Illinois at Chicago, United States of America

## Abstract

Recent genetic analyses have implicated several candidate susceptibility variants for schizophrenia. The single nucleotide polymorphism (SNP) rs7294919 is likely a schizophrenia-susceptibility variant according to its significant association with hippocampal volume, hippocampus function, and cognitive performance as well as the nominal association with schizophrenia. However, all previous analyses were conducted only in Europeans, and whether rs7294919 is associated with schizophrenia in other populations are yet to be tested. Here, we conducted a case-control analysis of rs7294919 with schizophrenia in six independent Chinese (N = 3) and Japanese (N = 3) samples, including a total of 7,352 cases and 10,824 controls. The results of our association analysis were not able to confirm the association of rs7294919 with schizophrenia (p = 0.51 in total samples, odds ratio = 1.02 for allele[C]). The absence of rs7294919’s association in Chinese and Japanese suggest a potential genetic heterogeneity in the susceptibility of schizophrenia on this locus and also demonstrate the difficulties in replicating associations of schizophrenia across different ethnic populations.

## Introduction

Schizophrenia is a complex psychiatric disorder with the lifetime prevalence of 1% in general populations worldwide. Family, twin and adoption studies have revealed a strong genetic component in schizophrenia with estimations of heritability about 80% [1]. So far, a great number of genetic studies on schizophrenia have been performed, and several susceptibility genes have been identified through linkage analyses [2], candidate gene association studies [3]–[5], and genome-wide association studies (GWASs) [6]–[8] etc. However, many of them cannot be successfully replicated across different populations, likely due to the already known genetic heterogeneity between different ethnic populations.

The hippocampal volume is a key brain structure for learning ability, memory process and stress regulation [9], and a reduction of hippocampal volume has been frequently reported in schizophrenia patients [10], [11]. In addition, the volume of hippocampus is also decreased in healthy relatives of schizophrenia patients, compared with healthy subjects in a recent large-scale meta-analysis of magnetic resonance imaging (MRI) studies [12]. These findings suggest that hippocampal volume is a plausible intermediate phenotype for schizophrenia [13], [14]. As expected, it has been demonstrated that many risk genes could affect hippocampal volume variations in schizophrenia patients, their healthy relatives as well as healthy controls [15]–[18].

Recently, two independent large-scale brain-imaging GWASs both identified a common genetic variant in rs7294919 located on Chromosome 12q24 being significantly associated with hippocampal volume in healthy subjects of European ancestry [19], [20]. Subsequently, in German samples, Erk et al. [21] observed a significant effect of rs7294919 in the right hippocampus showing that hippocampal activation increased with the number of the major alleles, and the same allele was also associated with decreased performance in a verbal learning and memory task, both of which are also considered to be intermediate phenotypes for schizophrenia [14]. These data implicated a possible association of rs7294919 with schizophrenia in Europeans, which was confirmed in a recent GWAS of schizophrenia in samples of European ancestry (p = 0.0285), although it did not reach the genome-wide significance level [7]. These convergent findings indicated that rs7294919 is likely a risk SNP for schizophrenia at least in Europeans.

However, whether rs7294919 is a risk SNP for schizophrenia in other populations is still unknown. Here, we attempted to test the association of this SNP with schizophrenia in multiple Han Chinese (N = 3, a total of 12,753 subjects) and Japanese (N = 3, a total of 5,454 subjects) case-control samples. Our results failed to replicate the association either in Han Chinese or in Japanese samples.

## Materials and Methods

### Han Chinese Schizophrenia Case-Control Samples

**Yunnan.** The Yunnan sample is comprised of 394 unrelated schizophrenia patients (mean age 38.5±10.4 years) and 305 unrelated healthy control subjects (mean age 35.4±12.5 years). The patients were recruited from The Second People's Hospital of Yuxi City and diagnosed as having schizophrenia by at least two psychiatrists according to ICD-10 criterion. Potential participants who have history of alcoholism, epilepsy, neurological diseases, or drug abuse were excluded. Meanwhile, unrelated healthy subjects were recruited from local community as control samples. These controls were all free from psychiatric disorders, drug abuse, alcohol dependence, or brain injury. All the patient and control subjects are of Han Chinese origin. This sample has been reported previously [22]–[25], and no population stratification was observed [3] when evaluated with the STRUCTURE software [26]. Genotyping of Yunnan sample was performed using SNaPShot as previously described [27], and the genotyping success rate was over than 99%.

**Singapore.** The Singapore sample (Han Chinese origin) has been described previously [28]. In brief, the Singapore sample is comprised of 882 unrelated schizophrenia patients (mean age 49.0±13.2 years) and 954 unrelated healthy controls (mean age 46.1±10.6 years). Schizophrenia patients were recruited from the Institute of Mental Health, Singapore, according to the DSM-IV criteria. Healthy volunteers were recruited from Singapore Prospective Study Program (SP2). All the patient and control subjects are of Han Chinese origin. Potential patient subjects with history of head injuries, substance induced psychotic disorders, alcoholic psychosis, or organic causes for psychoses were excluded. The healthy controls were all asked to provide detailed information about medical and family psychiatric histories. Healthy controls who had history of major mental disorders, alcohol or substance dependence, head injuries, or family history of psychiatric disorders were excluded. Genotyping in the Singapore sample was carried out using Illumina 1M genome-wide array according to manufacturer’s protocol, and the genotyping success rate was over than 99%. Test of population stratification in the Singapore sample using whole genome-wide SNPs found that the genomic control factor (λ) was 1.02, an indication of negligible population stratification.

**BIO-X.** The BIO-X sample has been reported in a GWA study in Han Chinese [8]. Briefly, the BIO-X sample included three datasets: the northern Han Chinese set of 1,578 cases and 1,592 controls recruited from Beijing and Shandong provinces; the central Han Chinese set of 1,238 cases and 2,856 controls recruited from Shanghai and Anhui provinces; and the southern Han Chinese set of 934 cases and 2,020 controls recruited from Guangdong and Guangxi provinces. All individuals with schizophrenia were interviewed by two independent psychiatrists, were diagnosed according to DSM-IV criteria, and had a 2-year history of the disease. Healthy controls were randomly selected from Han Chinese volunteers who were requested to reply to a written invitation to evaluate their medical history. Lists of potential control subjects were screened for suitable volunteers by excluding subjects with major mental illness. Population substructure was evaluated using principal-components analysis (PCA) using EIGENSTRAT software [29], [30]. Twenty components, some of which were predicted to reflect ancestry differences among subjects, were generated for each single sample. Logistic regression was used to determine whether there was a significant difference in component scores between cases and controls, and significant components were used as covariates in the association analysis to correct for population stratification. Genotyping in BIO-X sample was performed using the Affymetrix Genome-Wide Human SNP Array 6.0, and the genotyping success rate was over than 97%.

### Japanese Schizophrenia Case-Control Samples

**Aichi**. The Tokai sample has been used in a GWA study previously [31]. This sample included 575 unrelated schizophrenics (43.5±14.8 years) and 564 healthy controls (44.0±14.4 years). Patients were included if they 1) met DSM-IV criteria for schizophrenia; 2) were physically healthy and had normal routine laboratory tests; and 3) had no mood disorders, substance abuse, neurodevelopmental disorders, epilepsy, or mental retardation. Consensus diagnoses were made according to DSM-IV criteria by at least two experienced psychiatrists on the basis of unstructured interviews with patients and families and review of medical records. Control subjects were members of general public who had no history of mental disorders. This was ascertained during face-to-face interviews where subjects were asked if they had suffered an episode of depression, mania, or psychotic experiences or if they had received treatment for any psychiatric disorder. All included subjects were unrelated, living in the Tokai area of the mainland of Japan, and self-identified as Japanese. Written informed consent was obtained from each subject. Genotyping in the Aichi sample was conducted using the Affymetrix Genome-Wide Human SNP Array 5.0. After applying quality control criteria, the final Tokai sample consisted of 560 cases and 548 controls. Q-Q plots of the Aichi sample were generated on the basis of allele-wise analysis of SNPs that passed quality control, and the observed value of genomic control factor (λ) is consistent with those reported in well-matched samples (λ = 1.065).

**Niigata**. The Niigata sample is comprised of 662 patients (39.8±13.8 years) with schizophrenia diagnosed according to the DSM-IV criteria and 671 mentally healthy individuals (38.4±10.8 years), with no personal or family history (within first-degree relatives) of psychiatric disorders. A psychiatric assessment of each participant was conducted, as previously described [32]. All subjects were unrelated and of Japanese descent, and written informed consent was obtained from all participants. This sample has been repeatedly used in genetic association with schizophrenia [33]–[35], and was proved to be effective to identify potential risk variant. Although the status of population stratification was unknown in this sample, the cases and controls were collected very stringently from the same geographic area to minimize the potential population stratification. Genotyping of this sample was performed using TaqMan method, and the genotyping success rate was over than 99%.

**Tokushima**. The Tokushima sample consisted of 1,104 schizophrenia patients (57.0±14.3 years) and 1878 healthy controls (39.1±13.3 years). All subjects were biologically unrelated Japanese and were recruited at Tokushima [33]. Patients were recruited among both the outpatient and inpatient populations at university and psychiatric hospitals. Each patient with schizophrenia has been diagnosed by at least two trained psychiatrists according to DSM-IV criteria, based on an unstructured clinical interview. Controls were selected from volunteers who were recruited from hospital staff, students, and company employees documented to be free from psychiatric problems and past histories of mental illness. Written informed consent was obtained for all subjects after the procedures had been fully explained. Although this sample has not been tested for population stratification, the cases and controls were recruited from the same geographic location under the rigorous criteria of DSM-IV. In addition, this sample has been used in many large-scale genetic studies in Japanese [33], [36], [37], and demonstrated to be effective to detect genetic risk variants. Thus, it is expected that there should be no obvious population stratification in the Tokushima sample. Genotyping of this sample was performed using TaqMan method, and the genotyping success rate was over than 99%.

### Statistical analysis

The research protocol was approved by the internal review board of Kunming Institute of Zoology, Chinese Academy of Sciences. All the samples were analyzed under the appropriate ethical approvals, and written informed consents were obtained from all subjects or caregivers. Next of kin, care takers or guardians consented on the behalf of participants whose capacity to consent was compromised. Usually, patients with mild schizophrenia forms signed the consent, for moderate schizophrenia forms patients and care givers signed the consent and in severe schizophrenia forms care takers signed the consent. Logistic regression analyses were calculated firstly in each individual sample, and significance for the combined samples were assessed by meta-analysis using the Mantel-Haenszel method with fixed effects (inverse variance), as this is no indication of heterogeneity, which was conducted in the R package. Power analysis was performed by the Power and Sample Size Program software, and the commonly observed odds ratio (OR) of 1.10 was applied in the power analysis, which correspond to a ‘‘weak’’ gene effect.

## Results

We analyzed rs7294919 in six independent schizophrenia case-control samples from China, Singapore and Japan, with three of them being of Han Chinese origin (Yunnan, Singapore and BIO-X), and the other three samples are of Japanese ancestry (Aichi, Niigata and Tokushima). The detailed information (sample size, mean age, and genotyping method etc.) of the included samples is shown in **Table 1**. The SNP rs7294919 did not deviate from Hardy-Weinberg Equilibrium (HWE) in the six samples (p>0.1), and the frequency of minor allele [C] did not vary significantly among the tested control samples (0.16∼0.21, **Table 2**), which is consistent with the data from the 1000-Human-Genome project (∼0.23 in Asians).

**Table 1:** Characteristics of included samples on the association of rs7294919 with schizophrenia in Asians.

Population	Sample	Investigator	Schizophrenia Cases		Healthy Controls		Definition of schizophrenia	Genotyping method
			N	Mean Age	N	Mean Age		
Chinese	Yunnan	B Su	394	38.5±10.4	305	35.4±12.5	ICD-10	SNaPShot
	Singapore	J Liu	882	49.0±13.2	954	46.1±10.6	DSM-IV	Illumina 1M
	BIO-X	Y Shi	3,750	/	6,468	/	DSM-IV	Affymetrix 6.0
Japanese	Aichi	N Iwata	560	43.5±14.8	548	44.0±14.4	DSM-IV	Affymetrix 5.0
	Niigata	T Someya	662	39.8±13.8	671	38.4±10.8	DSM-IV	TaqMan
	Tokushima	S Numata	1,104	57.0±14.3	1878	39.1±13.3	DSM-IV	TaqMan

ICD-10, the international classification of diseases 10; DSM-IV, diagnosis and statistical manual of mental health disorders, fourth edition.

**Table 2:** Meta-analysis of rs7294919 with schizophrenia in our Asian samples.

Sample	Allele	N Cases	N Controls	Allele frequencies		P-value	Odds ratio	95%CI
				Cases	Controls			
Yunnan	C	394	305	0.1840	0.1623	0.28	1.17	0.88–1.55
Singapore	C	882	954	0.2098	0.1871	0.085	1.15	0.98–1.36
BIO-X	C	3,750	6,468	NA	NA	0.73	0.97	0.90–1.05
Chinese samples	C	5,026	7,727	NA	NA	0.78	1.01	0.94–1.08
Aichi	C	560	548	0.1973	0.1880	0.58	1.06	0.86–1.30
Niigata	C	662	671	0.1941	0.2042	0.52	0.94	0.78–1.14
Tokushima	C	1,104	1878	0.1993	0.1872	0.26	1.08	0.95–1.23
Japanese samples	C	2,326	3,097	0.1973	0.1910	0.45	1.04	0.94–1.14
Asian samples	C	7,352	10,824	NA	NA	0.51	1.02	0.96–1.08

CI, confidence interval; NA, not available.

Test of heterogeneity:

Chinese samples, p = 0.10; Japanese samples, p = 0.49; Asian samples, p = 0.27.

Rs7294919 was not associated with schizophrenia in any of the six samples, although the directions of OR for the minor allele [C] in Yunnan (1.17), Singapore (1.15), Aichi (1.06), and Tokushima samples (1.08) were similar with the data in the European samples (1.07) (**Table 2**).

Meta-analyses of rs7294919 in Chinese samples, Japanese samples, and the total Asian samples still indicated non-significant results (p>0.40), and the ORs in these samples are close to 1.00 (1.01 in Chinese, 1.04 in Japanese, and 1.02 in Asian), i.e., the effect size is rather small. Power analysis indicated that the present sample size (Chinese plus Japanese) revealed a >85% power of detecting a significant association of allele given an odds ratio of 1.10 (correspond to a weak gene effect), and the non-significant association is unlikely caused by the sample size. Therefore, rs7294919 is likely not a risk SNP for schizophrenia in Chinese or Japanese populations, which is different from the results in Europeans, suggesting there may exist genetic heterogeneity of this locus on the susceptibility to schizophrenia between different populations.

## Discussion

Rs7294919 is a recently GWAS identified SNP for hippocampal volume variation in populations of European ancestry [19], [20], and a subsequent functional MRI study further implicated its potential role in hippocampal activation [21], both of which have implied that rs7294919 may play an important role in hippocampal function and even in brain development. Interestingly, decreased hippocampal volume and aberrant hippocampal function have been consistently reported in schizophrenia patients compared with healthy controls [11], [38], and are considered to be intermediate phenotypes for schizophrenia. Therefore, rs7294919 is likely a reasonable risk SNP for schizophrenia based on these prior lines of evidence. As expected, rs7294919 also showed nominal significant association with schizophrenia in a recent GWAS of schizophrenia in Europeans (p<0.05), further confirmed the hypothesis that it is likely a risk SNP for schizophrenia.

However, all lines of the evidence were based on data from European samples, and the associations of rs7294919 with schizophrenia in other populations are still unknown. Therefore, in the present study, we selected two representative populations, Chinese and Japanese, which are commonly utilized in genetic association studies of schizophrenia, and tested the associations for rs7294919. In contrast to the results in Europeans, the SNP is not associated with schizophrenia in either of the Asian populations. Considering the relatively large samples we used, we propose that our current data do not support the speculation that rs7294919 is a risk SNP for schizophrenia in Chinese or Japanese populations.

Collectively, our genetic association data do not support the conclusion that rs7294919 is a susceptibility SNP for schizophrenia in Chinese or Japanese, and the inconsistencies are likely due to genetic heterogeneity between different populations.
